# Occurrence of Spot Blotch in Spring Barley Caused by *Bipolaris sorokiniana* Shoem. in South-Eastern Kazakhstan

**DOI:** 10.1155/2022/3602996

**Published:** 2022-08-26

**Authors:** Yerlan Dutbayev, Nurlan Kuldybayev, Saule Daugaliyeva, Elvira Ismailova, Nadira Sultanova, Göksel Özer, Ayana Slyamova, Kadyrzhan Mukin, Abdelfattah Dababat, Minura Yessimbekova

**Affiliations:** ^1^Kazakh National Agrarian Research University, Almaty, Kazakhstan; ^2^Institute of Microbiology and Virology, Almaty, Kazakhstan; ^3^Kazakh Research Institute of Plant Protection and Quarantine, Almaty, Kazakhstan; ^4^Faculty of Agriculture, Department of Plant Protection, Bolu Abant Izzet Baysal University, Bolu, Turkey; ^5^Kazakh Research Institute of Agriculture and Crop Production, Almalybak, Almaty, Kazakhstan; ^6^International Maize and Wheat Improvement Center (CIMMYT), Ankara, Turkey

## Abstract

In Kazakhstan, barley (*Hordeum vulgare* L.) is the second most important cereal crop after wheat, with an annual production of approximately 1.9 million tons. The study aimed to characterize *Bipolaris sorokiniana* isolates obtained from barley fields surveyed. A total of 21 diseased leaves showing spot blotch symptoms were collected from experimental plots located close to the Kazakh Research Institute of Agriculture and Crop Production, where the spring barley Arna cultivar was planted in June 2020. The overall strategy for control of spring barley blotch in the Almaty region of Kazakhstan should include the determination of the aggressiveness of the pathogen isolates to better understand the biology of the diseases and ultimately proper control strategy. Pathogenicity of *B. sorokiniana* isolates was made on barley seedlings in vitro. Inoculated seedlings showed clear symptoms of *B. sorokiniana*, and therefore, Koch's postulates were fulfilled by reisolating the pathogen from artificially inoculated seedlings and identifying it based on standard morphology criteria. Further investigation is needed to understand the impact of *B. sorokiniana* on barley production in Kazakhstan.

## 1. Introduction

In Kazakhstan, barley (*Hordeum vulgare* L.) is the second most important cereal crop after wheat, with an annual production of approximately 1.9 million tons. The average yield on an area of more than 1.5 million ha is approximately 1.3 tons/ha, which is almost three times less than the global barley production average [1].

As with any grain crop, barley production is affected by soilborne pathogens. *Cochliobolus sativus* Drechsler ex Dastur, anamorph *Bipolaris sorokiniana* (Sacc.) Shoemaker is a fungus in the Ascomycota division and one of the most important soilborne pathogens that causes root rot and spot blotch diseases in cereal crops, including wheat, barley, and triticale [2–6]. *B. sorokiniana* has wide geographic and host ranges [2, 3]. The low barley productivity in Kazakhstan is to a greater extent caused by the susceptibility of the cultivated varieties to spot blotch [7]. The pathogen infects barley crops and causes spot blotch, leaf spotting, ordinary stem and root rot, and “black seed,” and, ultimately, significantly reduces grain yield [8, 9].

Symptoms of *B. sorokiniana* in cereal rots include chlorosis and necrosis of cotyledons, water-soaked lesions on the crown and lower stem, stunting, preemergence and postemergence damping-off, wilting, and brown to black rot in the lower taproot and lateral roots with decay in the cortical region, and discoloration [10]. Estimating the impact of spot blotch on barley yield is not reliable because it frequently occurs in a disease complex, including numerous pathogens. The identification of the pathogenic species prevalent in a region is an important starting point for selecting an appropriate management strategy.

Recent studies devoted to studying *B. sorokiniana* in cereal diseases were carried out to reveal the population structure of leaf pathogens of spring wheat in Northern Kazakhstan [11] and the host compatibility of barley varieties to the pathogens of leaf spotting based on the artificial infectious background [6]. Most recently, the International Maize and Wheat Improvement Center (CIMMYT) conducted two intensive surveys on soilborne diseases in cereals in main cereal-growing areas in Kazakhstan [5, 12]. Their results showed that cereal-growing areas were severely infected with the soilborne disease and that *B. sorokiniana* was the most prevalent pathogen. Özer et al. [5] studied the biological characteristics of common root rot on triticale caused by *B. sorokiniana* and found that it caused growth retardation and internode necrosis on the roots. In another study carried out by Alkan et al. [13] in the Almaty region, the main pathogen isolates were identified as *B. sorokiniana,* while *Fusarium culmorum* and *Microdochium bolleyi* were also found to be less common, but pathogenic, through in vitro tests.

The best measure to control diseases is the use of resistant varieties, especially when these varieties are bred to have multidisease resistance to a complex of pathogens [14]. Crop rotation and presowing treatment of seeds with chemicals are still options where resistant varieties are unavailable [15]. In recent years, such disease symptoms have been frequently observed in barley in the Almaty region, with favorable conditions present earlier in the season.

This study aims to answer the following research question: how to characterize *B. sorokiniana* isolates obtained from barley fields surveyed? Therefore, the main objectives of the study were to isolate fungi from barley leaves showing spot blotch symptoms, identify obtained fungal isolates morphologically and by molecular techniques, and evaluate the pathogenicity of *B. sorokiniana* isolates on barley seeds associated with spot blotch of spring barley in the Almaty region of South-Eastern Kazakhstan. The study consists of five sections, namely, the Introduction, which includes the literature review, Materials and Methods, which include research design and stages of the study, Results, Discussion, and Conclusion.

## 2. Materials and Methods

### 2.1. Research Design

To achieve the objectives of this study, the spring barley Arna cultivar was planted in early April 2020 on 10 m^2^ experimental plots (three replications) (43.237589°N, 76.692629°E). A total of 21 diseased leaves showing spot blotch symptoms were randomly collected at the end of June 2020 in the laboratory of the gene fund of field crops of Kazakh Research Institute of Agriculture and Crop Production. The sampled leaves were put into boxes placed in appropriate containers and immediately transferred to the laboratory of Kazakhstan-Japan Innovation Center of Kazakh National Agrarian Research University in Almaty. This condition was also done for the determination of pathogenicity test of the two isolates of *B. sorokiniana* according to Koch's postulates. At the first stage of the study, we carried out pathogen isolation using generally accepted microbiological methods and identification using the ITS region sequencing method. In the second phase of the study, a pathogenicity test was performed using a modified method by Broders et al. [16]. At the last stage of the study, a statistical analysis of the results was carried out.

### 2.2. Stages of the Study

#### 2.2.1. Pathogen Isolation and Identification

To isolate *B. sorokiniana* isolates, the pieces of barley leaves showing symptoms were surface sterilized in 90% ethanol for 1 min, followed by immersion in a 1% sodium hypochlorite solution for 3 min and by three rinses in sterile water. The symptomatic leaf tissues of 5 mm^2^ were excised from representative necrotic spots and placed on a 1/5 strength potato dextrose agar (PDA) amended with 0.01% streptomycin. After 5 days of incubation at 25°C in the dark, fungal colonies were observed and transferred to fresh PDA plates using the single-spore technique. Fungi were routinely grown on PDA at 25°C. All isolates were stored at 4°C on PDA stock plates and on filter papers in the microcentrifuge tubes at −20°C.

Morphological and cultural characteristics of the dematiaceous fungal isolates were identified by following the key of the Institute of Microbiology and Virology of the Ministry of Education and Science of the Republic of Kazakhstan, using a light microscope (Premiere, Ningbo ZHANJING Optical Instrument Co., China) at 200× magnification [17, 18].

Isolates of *B. sorokiniana* were grown on Saburo agar, potato dextrose agar (PDA), and Czapek-Dox medium [3] and were kept in an incubator for 5 days at 25 ± 1°C [4]. The main morphological parameters, such as conidial length, width, and quantity of sept, were measured from 50 conidia for each isolate [19]. To obtain pure colonies, at least 10 spores were transferred and cultured at room temperature (about 25°C) [20]. In order to minimize the risk of loss of pathogenicity and to make the initial inoculum available for further subculturing, the pure culture was stored at 4°C on a PDA tilt.

Molecular identification of the fungal species was performed by sequencing the ITS region as per the Sanger method [21]. To extract DNA, mycelia were harvested from 3-day to 7-day PDA cultures, then frozen at −20°C, and grounded with a pestle in a 1.5 ml Eppendorf tube to a powder state and subjected to the Fungi DNA Isolation Kit (Norgen Biotek Corp., Ontario, Canada) according to the manufacturer's protocol. The DNA concentration in the samples was determined using a QubitTM dsDNA HS assay kit fluorimeter (Life Technologies, Oregon, USA) on a scale for dsDNA HS.

The universal primers ITS1/ITS4 were employed to amplify the ITS region of ribosomal RNA. The amplification reaction mixture was composed of 12.5 *μ*l Q5® Hot Start High-Fidelity 2 × Master Mix (New England BioLabs, MA, USA), 1.25 *μ*l of each primer (10 *μ*M), 1.5 *μ*l DNA (10 ng/*μ*l), and 8.5 *μ*l sterile distilled water. PCR amplification was carried out in an Eppendorf ProS amplifier (Eppendorf, Hamburg, Germany). PCR amplification mode included an initial denaturation at 94°C for 10 min, 30 cycles at 94°C for 30 s, 55°С for 1 min, 72°С for 40 s, and followed by a final extension step at 72°С for 10 min. The amplification results were analyzed on a 1.2% agarose gel and purified using CleanSweep™ PCR Purification reagent (Applied Biosystems, USA).

The sequencing reaction was carried out using the BigDye Terminator v3.1 Cycle Sequencing Kit (Applied Biosystems, USA) according to the manufacturer's instructions (BigDye® Terminator v3.1, Cycle Sequencing Kit Protocol, Applied Biosystems, USA), followed by fragment separation, using the 3500 DNA Analyzer-automated genetic analyzer (Applied Biosystems, USA). The sequencing results were processed in the SeqA program (Applied Biosystems, USA). The obtained nucleotide sequences of the ITS region were compared with the data of the GenBank database using the BLAST program [22]. Phylogenetic analyses were performed using MEGA X software [21]. The alignment of nucleotide sequences was carried out using the ClustalW algorithm. To build phylogenetic trees, the neighbour-joining (NJ) method was used.

#### 2.2.2. Pathogenicity Test

The determination of pathogenicity of the two isolates of *B. sorokiniana* was carried out at the Kazakhstan-Japan Innovation Center of Kazakh National Agrarian Research University in Almaty. The virulence assessment degree of the fungal isolates was fulfilled with the pathogenicity test according to the modified method of Broders et al. [16]. The Arna cultivar that adapted to the conditions of South-Eastern Kazakhstan was employed for pathogenicity assays. The seeds of the cultivars used in the pathogenicity test and leaves were not included.

Conidia were harvested by adding distilled water to the 5-day-old PDA plates and scraping the agar surface with a spatula. This conidial suspension was removed from mycelial fragments by filtering it through two layers of cheesecloth. The inoculum concentration was adjusted to 8 × 10^3^ conidia per ml with distilled water using a hemacytometer.

For inoculation, the seeds were immersed in the fungal suspension for 10 min and then left to dry. Evenly distributed seeds were laid out on Petri dishes of water agar in three replicates. The barley seeds were placed at a distance of 1.5–2 cm from each other in aseptic conditions in a moist chamber (high humidity). There was a total of 20 barley seeds in each chamber. Then, seeds were incubated at 25°C in a thermostat until the fungal mycelium was well grown after 7 days in the darkness. After this period, the germination of the disease was assessed according to a 0–3 scale as per Broders et al. [16], where 0 = 100% germination rate without disease symptoms of seeds (roots) infection; 1 = 70–99% germination with root-lesion formation; 2 = germination of 30–69% with coalesced lesions; and 3 = 0–29% germination, where all seed tissues were affected. During the experiment, all samples were regularly examined and fixed. Eight seeds were placed per Petri dish containing PDA supplemented with 0.01% tetracycline (PDAt). The agar plates were placed in an incubator for 7 days at 25°C [10].

#### 2.2.3. Data Analysis

Statistical processing was performed using the RStudio-integrated development environment. The significance for all variables for parametric data was performed with a nonparametric Kruskal–Wallis one-way analysis of variance with the *P* value [23]. We evaluated two work hypotheses:

  Hypothesis 1:
 
*H*_0_: the differences between length, width, and the number of septa of P-08 and P-15 isolates are equal;
 
*H*_*A*_: the differences between length, width, and the number of septa of P-08 and P-15 isolates are not equal.
  Hypothesis 2:
 
*H*_0_: the infection factor (the infection from P-08, P-15 *B. sorokiniana* isolates and control, without infection) cannot impact spot blotch development and incidence on barley seedlings;
 
*H*_*A*_: the infection factor (the infection from P-08, P-15 *B. sorokiniana* isolates and control, without infection) can impact to spot blotch development and incidence on barley seedlings.

## 3. Results

**Figure 1:**
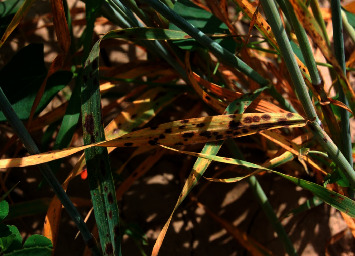
Symptoms of spot blotch on spring barley leaves from the surveyed experimental plots in this study.

A total of 11 fungal isolates were obtained from symptomatic spring barley samples collected from experimental plots of the Kazakh Research Institute of Agriculture and Plant Growing in the Almaty region in 2020. The field samples of barley leaves with symptoms of spot blotch are shown in Figure 1.

Species identification based on morphological keys and the sequencing of the ITS region showed that two isolates (P-08 and P-15) were *B. sorokiniana*. The remaining nine isolates were identified as *Alternaria alternata* (three isolates), *A. tenuissima* (one isolate), *A. infectoria* (two isolates), *Lecanicillium aphanocladii* (two isolates), and *Cladosporium* sp. (one isolate).

**Figure 2:**
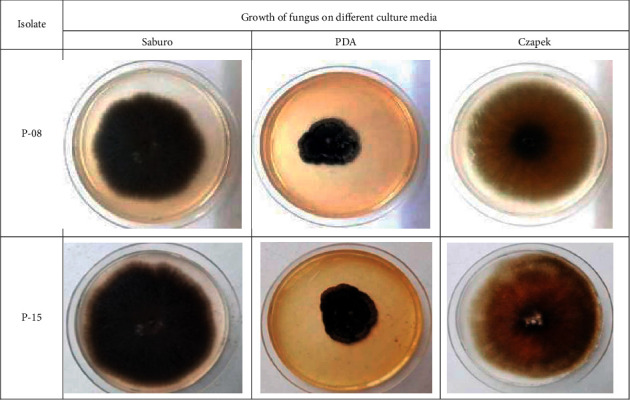
Cultures of the two identified *B. sorokiniana* isolates cultured on Saburo, PDA, and Czapek media.

The growth of the P-08 and P-15 isolates of *B. sorokiniana* was evaluated on Saburo, PDA, and Czapek artificial cultural media at 25°C. The fungal isolate growth on Saburo at 25°C media colony was fast, rounded, and outstretched, at first olive-colored and later turning black. The consistency was fluffy and woolly with a slightly raised color. The growth of colonies on Czapek media was fast, rounded, and outstretched, at first olive-colored and later turning black. The consistency was fluffy and woolly with a slightly raised color. The isolate colonies were grown quickly on PDA with rounded and outstretched, at first olive-colored and later becoming dark grey with an almost black core. The mycelium was fluffy and woolly. The edges were slightly wavy (Figure 2).

**Figure 3:**
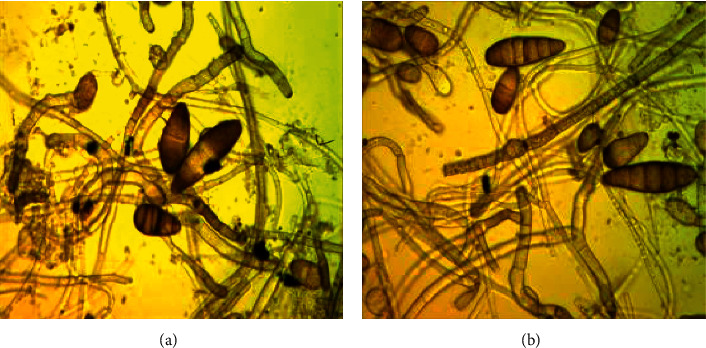
Conidia of *B. sorokiniana* isolates Р-08 (a) and Р-15 (b) (×400).

Five days after incubation at 25°C in the dark, fungal colonies were observed, and those with similar morphological features were cultured on new PDA plates, using the single-spore isolation technique. The mean conidium length, width dimensions, and the number of septa (*n* = 50) for *B. sorokiniana* isolates: isolates were formed at the end, olive-brown, elliptical, with pointed ends-11–28 × 58–142 *μ*m with 4–9 septa slightly curved, the conidial wall was smooth with thickenings on the septa. The conidiophores were mostly single or grouped, simple, cloisonne, erect, 3–9 × 100–142 *μ*m. There were no significant differences between the isolates (Figure 3).

**Figure 4:**
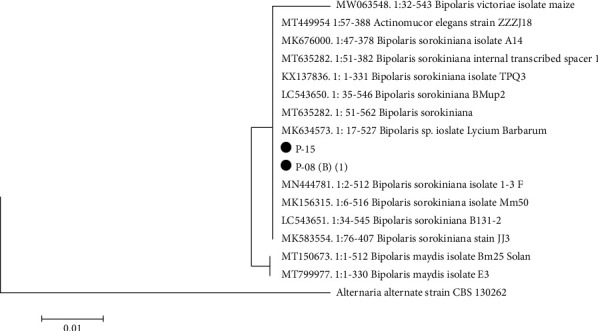
Phylogenetic tree for representative *B. sorokiniana* isolates.

The phylogenetic tree of P-08 and P-15 isolates of *B. sorokiniana* was constructed by comparing the ITS region of the sample under study with the sequences of reference strains derived from GenBank [22]. The degree of homology with the closest strain MN444781: 2–512 and *B. sorokiniana* isolate 1–3 F was 100% (Figure 4).

**Figure 5:**
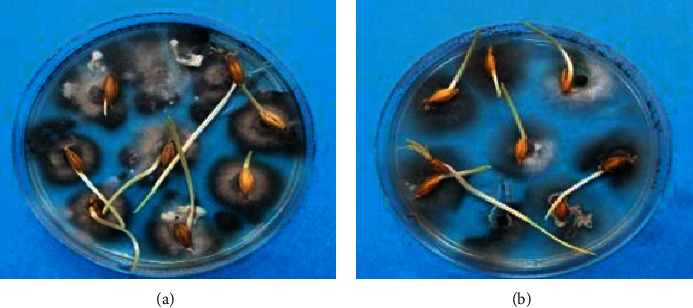
The display of pathogenicity of the two *B. sorokiniana* isolates on barley seeds (cultivar Arna). (a) P-08 isolate; (b) P-15 isolate.

The two isolates caused symptoms on spring barley seedlings, including necrosis and discoloration of plant roots and seedlings (Figure 5, Table 1). Koch's postulates were fulfilled by reisolating and identifying the *B. sorokiniana* pathogen based on the morphology described above. The mean disease incidence was 100% for both the isolates. The germination scores ranged at 71.5% for the P-08 isolate and 48.5% for the P-15 isolate and were significantly different (<0.0001) in terms of their development (Table 2). No disease symptoms developed on control barley seedlings.

## 4. Discussion

The current study focused on isolates of *B. sorokiniana* obtained from spring barley associated with spot blotch in the Almaty region of Kazakhstan. Eleven isolates from diseased leaves of barley were identified as *B. sorokiniana* (two isolates), *Alternaria alternata* (three isolates), *A. tenuissima* (one isolate), *A. infectoria* (two isolates), *L. aphanocladii* (two isolates), and *Cladosporium* sp. (one isolate).

*B. sorokiniana* was obtained from the leaf tissues of barley. Based on the traditional methods of fungal isolation from leaf spot-affected tissues of spring barley [5], two isolates P-08 and P-15 demonstrating the external signs of mycelium characteristic of these media and differing in the time of their growth were identified. The most distinctive growing medium for the pathogen was potato dextrose agar, where it exhibited its rounded/outstretched characteristic, olive-colored at the beginning and then becoming dark grey with an almost black core. Similar signs of mycelium were noted in Verma et al. [24], where some patterns were blackish grey with a whitish fluffy area. A feature of the PDA medium is also the ability to form the largest number of *B. sorokiniana* spores/ml [20].

The conidium sizes of the *B. sorokiniana* in barley examined in this study were consistent with that description from Sivanesan [18] and showed similar results to those obtained in previous studies by Samuels and Sivanesan [25], Agrios [2], and Özer et al. [5, 6]. The conidium characteristics were useful criteria for the discrimination of *B. sorokiniana* isolates from other isolates due to their morphological similarities [5, 6, 25].

In the current study, the ITS sequences of the isolates were matched with reference sequences of *B. sorokiniana* in GenBank. The species specificity of *B. sorokiniana*, supported by their morphological parameters, has allowed researchers to apply PCR-based molecular techniques, such as microsatellite analysis [26], RAPD [27], and ITS regions [24]. The latter method made it possible to accurately determine the most aggressive isolate of spot blotch pathogen from the infected leaf and seed samples of wheat [28]. However, some authors [29] questioned the accuracy of this method in terms of phylogenetic information content within the genus *Gibberella* and the lack of species. However, according to a phylogenetic tree, the identified isolates P-08 and P-15 are part of the *Bipolaris* clade that clustered with other nine *Bipolaris* isolates including some related species as *Actinomucor elegans* strain ZZZJ18 and *Bipolaris victoriae* isolate on maize. Also, Meng et al. [30] found that this technique of identifying and orthologous clustering allows classifying and assigning some core and single-copy genes of *Bipolaris* genomes.

In the present work, the infective nature of both *B. sorokiniana* isolates obtained from diseased spring barley leaves was confirmed by means of a pathogenicity test on barley seedlings with distinct symptoms of necrosis and discoloration. However, in terms of having a mean disease index of pathogen development, the P-15 isolate showed greater aggressiveness (71.5%) compared to that of the P-08 isolate (48.5%) in the artificial background. Barley seeds without infection (not inoculated) showed no symptoms of the disease.

The aggressive nature of this pathogen can vary significantly. Kumar et al. [31] found that 22 conidial descendants of the 25 most aggressive conidia of *B. sorokiniana* inoculated on barley caused necrotic lesions on the third day, while the remaining three conidia caused necrotic lesions on the fourth day. The demonstration of varying degrees of *B. sorokiniana* aggressiveness is directly related to the environment, especially in hot and humid climates [32]. Knight et al. [33] assessed 31 *B. sorokiniana* isolates for their ability to cause spot blotch infections on barley leaves using a differential set of 15 barley genotypes and three other cereal species. The 14 isolates are from crown root rot infections of either wheat or barley and 14 isolates from spot blotch infections of barley. Phenotypic experiments revealed that isolates of *B. sorokiniana* collected from barley spot blotch infections had a high level of pathogenic variability on inoculated barley seeds.

This study also is the first report of *B. sorokiniana* causing spot blotch on spring barley in South-Eastern Kazakhstan.

## 5. Conclusion

This study aimed to isolate, identify, and evaluate the pathogenicity of *B. sorokiniana* isolates associated with spot blotch in a spring barley cultivar in the Almaty region of South-Eastern Kazakhstan. The two isolates of *B. sorokiniana* were identified as agents for spot blotch in barley. Saburo, PDA, and Czapek artificial cultural media were used for the culturing of P-08 and P-15 isolates of *B. sorokiniana* and were optimal for their growth. The biological features of P-08 and P-15 conidia, including the length, width, and the number of septa, were described. In addition, several fungal species were obtained from single-spore cultures (monosporous cultures) and were identified as *Alternaria alternata*, *A. tenuissima*, *A. infectoria*, *Lecanicillium aphanocladii*, and *Cladosporium* sp.

Isolates of *B. sorokiniana* were able to infect and colonize barley seedlings to a high degree. The pathogenicity assays fulfilled Koch's postulates. The isolates without infection did not demonstrate disease symptoms. Further investigation is needed to understand the impact of *B. sorokiniana* on barley production in Kazakhstan. Future research needs to focus on studying *B. sorokiniana* isolates in barley photosynthesis processes.

## Figures and Tables

**Table 1:** Structural parameters of two isolates of *B. sorokiniana* on Czapek artificial cultural media barley seeds.

Isolate	Conidia of *B. sorokiniana*		
	Length, *μ*m	Width, *μ*m	Septa
P-08	105.7	16.6	6.2
P-15	93.7	14.6	5.9
ANOVA, *P* value	0.178	0.098	0.189

**Table 2:** The spread and development of symptoms in barley seeds in the pathogenicity test of two isolates of *B. sorokiniana* (PDA, at 23–25°C, KazNARU, 2021).

Isolate	Disease index of barley seeds, KazNARU, 2021 (%)	
	Incidence	Development
P-08	100	71.5
P-15	100	48.5
Control (without infection)	0	0
*P* value (nonparametric Kruskal–Wallis test)	<0.0001	

## Data Availability

The data used to support this study are available from the corresponding author upon request.
